# Hyperlipidaemia and Weight Amongst Afghani Refugees Attending a General Practice Clinic in Regional Australia

**DOI:** 10.1007/s10903-022-01446-1

**Published:** 2023-02-06

**Authors:** S. P. Mehdi, J. Pasricha, B. A. Biggs

**Affiliations:** 1 Tristar Medical Group, Mildura, Australia; 2 Royal Melbourne Hospital, Victorian Infectious Diseases Service, Melbourne, Australia; 3 Peter Doherty Institute for Immunity and Infection, 792 Elizabeth St, Melbourne, VIC 3000 Australia

**Keywords:** Refugee, Immigrant, Afghanistan, Screening, Immunisation

## Abstract

Obesity and chronic disease are increasing problems in refugee populations. Afghani refugees undergoing hepatitis B vaccination between January 2015 and December 2017 at a general practice clinic received dietary counselling sessions in Dari by native speaking clinicians. Anthropometry, blood pressure, fasting lipids and liver function tests were measured at both visits with results compared over time. 110/119 refugees requiring hepatitis B vaccination were recruited into the study. Mean BMI was lower at follow up visits (25.4 vs 26.1 by wilcoxon signed rank test p 0.04) with 72 of 110 participants losing a median of 2 kg between visits (range 0.5–14 kg) a mean of 206 days after the initial consultation. Median triglyceride levels were lower at the second visit than the first (1.4 vs 1.3 mmol/L Wilcoxon signed rank test Z = 3.5, p 0.0004). This cohort of refugees lost weight and showed a small improvement in triglyceride levels between visits.

**Supplementary Information:**

The online version contains supplementary material available at 10.1007/s10903-022-01446-1.

## Introduction

Australia receives approximately 20,000 refugees per year [1]. In 2019 there were an estimated 15,000 Afghani nationals permanently resettled in Australia with a further 3500 residing under a temporary protection visa [1].

Post-settlement, refugees are particularly vulnerable to rapid weight gain [2–4], obesity and obesity-related disease [5, 6]. Whilst the causes of this vulnerability are not fully understood, several factors contribute. Firstly, food insecurity and malnutrition are common amongst refugee populations. Afghanistan has one of the highest levels of food insecurity and malnutrition in the world with 43% of Afghani children having low height for age (stunting) [7]. Malnutrition whilst in-utero or during infancy is associated with several biological effects that preference accumulation of fat over lean body mass. When food becomes readily available, this leads to higher risk of adiposity and central obesity as an adult [8]. In addition, food insecurity alters food preferences and consumption behaviours predisposing to weight gain [9]. Secondly, severe trauma such as conflict and forced migration increases susceptibility to hypertension, diabetes mellitus and cancer [10]. Thirdly, the challenges of settlement have detrimental effects on diet, activity levels [11] and health behaviours such as accessing healthcare. Finally, migration results in a loss of gut microbiome diversity and function which are associated with the development of obesity [12].

Obesity and obesity related diseases are important and growing problems amongst refugee populations. The characteristics and extent of this problem amongst Australian refugees has not been adequately determined and the effectiveness of preventative strategies in this group is unknown.

## Methods

Tristar medical group is a busy general practice in Mildura, Victoria which serves a large population of Afghani refugees settled in the region. Two general practitioners and many of the nurses working in the clinic are of Iraqi and Afghani background and are fluent in Dari, one of the main languages spoken by their Afghan refugee patients.

We aimed to measure the BMI and other nutritional parameters amongst refugees taking part in a hepatitis B vaccination programme at Tristar Medical Clinic from 2015 to 2017. Refugees were screened to assess whether they were hepatitis B immune and if non-immune or partially immune asked to return for hepatitis B vaccinations. All refugees being tested to assess hepatitis B immunity received a short, structured dietary counselling session, underwent a short questionnaire and had anthropometric and laboratory measures taken. Refugees identified as needing a full course of vaccination (3 doses over a six-month period) had repeat measurements taken at the time of their third dose of hepatitis B vaccination whilst those with indeterminate hepatitis B surface antibody levels (< 12 IU/mL) and requiring only booster vaccinations had repeat measurements taken at the time of receiving their vaccination. Therefore the entire cohort received a counselling session and had nutritional parameters measured but only some returned for repeat measures at 6 months (if receiving a full course of vaccinations) or at varying times (if receiving booster vaccinations only).

The counselling session was developed by two GP’s at the practice and covered the importance of nutrition, healthy food groups and portion sizes. The session was brief, structured and delivered in a standardized way using a visual aid (see Online Appendix). Counselling was in Dari and provided by two GPs who are native speakers, of similar cultural background to their refugee clients and who provide regular clinical care for most of the participants. All refugees completed a short questionnaire on demographic and lifestyle information and had their weight, height, waist circumference and blood pressure measured. Lipid studies and liver function tests were performed.

Measurements and questionnaire results were recorded in an excel database by one clinician. STATA SE 16 was used for the statistical analysis. Descriptive analyses were performed. Changes in BMI, lipid profile and LFT over time were compared using paired t-tests for normally distributed variables, wilcoxon signed rank tests for variables that did not follow a normal distribution and McNemar tests for categorical variables. Uni- and multi-variate linear (for continuous variables) and logistic (for categorical variables) regression analyses were performed to look for associations between body mass index and demographic/lifestyle factors such as age, sex, education level and visa status. Regression analyses were also performed to look for associations between weight loss and demographic/lifestyle factors. Results were considered to be statistically significant if the p value < 0.05. Variables were selected a priori based on clinical consideration and previous literature.

### Ethics Review

This project was reviewed and granted approval by the Human Research and Ethics Committee at Melbourne Health (HREC 2019.212).

## Results

From January 2015 to December 2017 230 refugees attending the clinic were identified as having unknown hepatitis B immunity. All 230 (100%) agreed to participate in the study. The majority were male (63.4%) with a mean age of 34.2 years (range 7–68.5 years) and had received limited education (30.1% none, 34.3% primary level). Most were permanent Australian residents (58.9%) or citizens (33.6%) and had been living in Australia for a mean of 6.2 years (range 3 months to 18 years). Demographic characteristics are summarized in Table 1.

**Table 1:** Demographic characteristics of Afghani refugees (n = 229)

Variable	Mean	SD	Min	Max
Age	34.2	16.1	7.4	68.4
Time in Australia (years)		4.9 (3.7)	.03	18

	N (%)			
Sex				
Male	146 (63.5)			
Female	84 (36.5)			
Education				
None	64 (30.1)			
Primary	73 (34.2)			
Secondary	53 (24.9)			
Tertiary	8 (3.8)			
Missing	15 (7.1)			
Visa category				
Australian citizen	73 (33.6)			
Permanent residency visa^1^	128 (59.0)			
Temporary Protection^2^ Visa	13 (6.0)			
Temporary Residency visa^3^	1 (0.5)			
Missing	2 (0.9)			
Smoking				
Yes	17 (7.9)			
No	198 (92.1)			

^1^Holders of this visa have the right to remain in Australia however they are unable to vote, do not have automatic right of entry should they travel overseas (and must obtain an authorisation to travel) and are not eligible for all government services and benefits that a citizen does

^2^This visa is for individuals who arrive in Australia without a visa and want to seek asylum. It allows the holder to stay in Australia temporarily for 3 years. The holder is able to work and study and to access government services

^3^Holders of this visa are residing in Australia temporarily usually for work purposes. Temporary residents do not have access to government services including healthcare

119/230 (51.7%) refugees screened were identified as needing hepatitis B vaccination being either hepatitis B non-immune (116/230) or having indeterminate hepatitis b antibody levels (< 12 iu/mL) (3/230). Of these, 110/119 (92.4%) returned for repeat vaccinations and had repeat measurements taken.

The mean BMI at baseline was 26 (range 18.2–61.3) with 53.3% of adults (age ≥ 18 years) and 19.1% of children (age < 18 years) in the overweight/obese BMI range. 86.4% of participants had an increased waist circumference (> 90 cm for males and > 84 cm for females) and 19.5% (45/230) were hypertensive (SBP > 130, DBP > 85). ‘Dys-lipidaemia’ defined as any of elevated triglyceride, elevated low-density lipid (LDL) level or reduced high-density lipid level (HDL) was present in 43.9% of refugees. Abnormalities in liver enzymes (any of increased ALT, ALP, AST, GGT or Bilirubin) were found in 47.1% (96/230) of participants. These findings are summarized in Table 2.

**Table 2:** Results of liver function tests and cholesterol levels at first and second clinic visits

Test	First visit	Second visit	Test of difference^a^
	Median/mean (range)	Median/mean (range)	
*Alanine aminotransferase (ALT)* U/L	20.0 (7–98)	21.0 (7–83)	0.68
*Aspartate transaminase* (AST) U/L	20.0 (12–79)	19.0 (11–43)	0.31
*Alkaline phosphatase* (ALP) U/L	79 (43–566)	79 (38–398)	0.50
*Gamma glutamyl transferase* (GGT) U/L	17 (6–124)	20 (5–143)	0.27
Bilirubin	9.0 (2.0–54)	10.0 (2.0–34)	0.18
Triglycerides mmol/L	1.4 (0.3–5.5)	1.3 (0.4–4.6)	0.0004
High-density lipoprotein (HDL) mmol/L	1.3 (0.7–2.4)	1.2 (0.6–3.2)	0.52
Low-density lipoprotein (LDL) mmol/L	2.7 (0.2–4.9)	2.6 (0.7–5.0)	0.09
Body Mass Index^b^	26.3 (18.5–40.4)	25.4 (18.4–39.3)	0.003
Weight (kg)	70.8 (47–105)	70.2 (47–101)	0.03
Systolic BP (mmHg)	118.1 (88–160)	118.7 (81–177)	0.84
Diastolic BP (mmHg)	73.7 (49–96)	74.5 (45–102)	0.39

^a^Wilcoxon signed rank test (non-parametric data) or paired t tests (parametric data)

^b^BMI calculated as weight in kg/(height in metres)^2^

110 of 119 refugees identified as needing hepatitis B vaccinations returned for repeat doses of hepatitis B vaccine 10–275 days following the first (mean 206 days). At these follow up visits, the mean BMI of refugees was lower than at the first visit (25.4 vs 26.1 wilcoxon signed rank test p 0.04) with 72 of 110 participants losing a median of 2 kg between visits (range 0.5–14 kg). Furthermore, median triglyceride levels were lower amongst refugees at the second visit than the first (Wilcoxon signed rank test Z = 3.5, p 0.0004). There were no significant changes in blood pressure, HDL or LDL cholesterol levels or liver function between visits.

On multivariate logistic regression, having a physical job and cooking for oneself were associated with weight loss (Table 3) and on multivariate regression, a body mass index in the overweight/obese range was associated with age, educational attainment and length of time living in Australia and was negatively associated with regular physical exercise. These findings are summarised in Table 4.

**Table 3:** Results of uni- and multi-variate regression modelling of factors associated with weight loss

Variable	Category	Odds ratio	p value
Age		1.02 [1.01–1.04]	0.004
Sex		1.38 [0.77–2.47]	0.275
Education	None	reference	
	Primary	0.76 [0.38–1.51]	0.436
	Secondary	0.53[0.25–1.13]	0.105
	Tertiary	0.81 [0.18–3.66]	0.787
Visa	Australian citizen	Reference	
	Permanent resident visa	0.78 [0.43–1.42]	0.428
	Temporary protection visa	0.71 [0.20–2.54]	0.239
	Temporary resident visa	1	
Physical job		2.46 [1.21–5.02]	0.013
Consumption of 2 serves of fruit per day		1.1 [0.62–1.96]	0.742
Consumption of 4 serves of vegetables per day		0.87 [0.429–1.76]	0.702
Car ownership		0.94 [0.48–1.81]	0.850
Daily exercise for 30 min		0.93 [0.51–1.67]	0.928
Does own cooking		1.61 [1.12–2.22]	0.004

**Table 4:** Results of uni- and multi-variate regression modelling of associations between BMI and other variables in 229 Afghani Refugees attending a regional GP clinic

Univariate regression				Multivariate regression model	
Variable	Categories	Coefficient	p value*	Coefficient	p value
Age		0.12	< 0.001	0.11	< 0.001
Male sex		0.19	0.78		
HDL		− 4.82	< 0.001		
LDL		1.64	< 0.001		
Triglycerides		1.29	< 0.001		
Waist circumference		0.25	< 0.001		
Abnormal LFT		1.43	0.037		
Education	*None*	2.69	< 0.001	0.39	0.591
	Primary	0.75	0.287		
	Secondary	− 1.25	0.102		
	Tertiary	0.65	0.718		
	Missing	− 5.11	< 0.001		
Visa	Citizen	1.75	0.012		
	Permanent resident	− 0.87	0.188		
	Temporary protection visa	− 0.92	0.531		
	Temporary resident	− 1.07	0.829		
	Missing	− 0.75	0.880		
Time in Australia		0.42	< 0.001	0.33	< 0.001
Five serves of fruits per day		− 0.98	0.157		
Two serves of vegetable per day		0.07	0.928		
30 min physical activity per day		− 1.97	0.005	− 1.47	0.021
Owns a car		0.82	0.305		
Physical job		0.50	0.569		

*A p value of < 0.05 was taken to be statistically significant

## Discussion

Refugees in our study had stable or modestly reduced BMI and triglyceride levels when attending follow up appointments approximately six months after their initial visit and counselling session. This is surprising as most studies find that refugees gain weight steadily following settlement in their host country [3, 4, 13, 14]. Longitudinal studies of refugees in the USA found them to be slimmer than the general American population on arrival but to gain weight rapidly (23% risk of overweight/obesity per year) such that overweight/obesity amongst refugees exceeded that of the host population at10 years post arrival [4]. A similar phenomenon is seen in immigrants [15] however the rate of weight gain in immigrants is slower than in refugee populations and rates of overweight/obesity in immigrants approximate rather than exceed the host population. Additionally migrants have lower rates of metabolic disease compared to non-migrants [16] despite their increasing BMI (“the healthy immigrant effect”) whereas refugees have increased rates of obesity-related chronic disease compared to non-refugees with some studies finding a greater burden of obesity-related disease than would be expected for the BMI suggesting that weight gain potentially confers greater metabolic risk in this population than in others [17]. These differences between refugees and migrants suggests there may be factors in the refugee experience beyond migration that increases risks of obesity and obesity-related disease.

Ethnic heterogeneity is consistently found in studies of refugee weight gain with South-east Asians and Africans experiencing the greatest increases in overweight/obesity over time. Middle Eastern refugees tend to have higher BMI’s at settlement but slower weight gain over time and, in the case of Middle Eastern women, weight loss [4]. We found a lower rate of obesity than that described in studies of Middle Eastern refugees in the USA [4]. There is little data on obesity rates in Afghanistan, however, the World Health Organization estimate 15% of Afghani’s to be overweight/obese [18]

Weight gain in refugees has been predominantly described in the USA [3, 4, 13] and whether it applies to refugees living elsewhere is less understood. In South Korea, North Korean refugees who defected recently (< 5 years) had half as much obesity as the South Korean population but those settled for ten years or more had the same prevalence of obesity as the host population. North Korean refugees had greater central obesity throughout, including at earlier timepoints when their BMI was lower. North and South Koreans share genetic backgrounds, diet and lifestyle yet North Korean refugees still underwent rapid weight gain and had greater central obesity and increased risk of metabolic disease when compared to South Koreans [19]. This finding suggests that increased obesity and metabolic diseases amongst refugee populations is not solely attributable to genetic differences between refugee and host populations or to dietary and lifestyle changes following settlement.

A complex relationship between overweight/obesity, malnutrition and food insecurity exists in refugees as demonstrated by a survey of 2005 Western Sahara refugee households living in camps in Algeria. There were almost equal amounts of obesity (20%), stunting/malnutrition (25%) and double burden obesity and malnutrition (25%) [20]. A similar pattern of co-existent obesity and malnutrition was found in a study of Afghani refugee households in Iran [21]. Prolonged mild food insecurity results in the paradoxical coexistence of underweight and overweight in a population [22] with women being particularly susceptible to obesity following prolonged periods of food insecurity in both developing and developed world settings.

It is becoming increasingly clear that malnutrition during critical periods of early development (particularly in-utero and infancy) has profound effects on metabolic handling as an adult. Malnutrition causes changes in organogenesis hormonal axes regulating growth and appetite, gene expression, telomere length and maturation of the gut microbiome. Collectively, these biological changes shift the handling of metabolic loads towards adiposity and the metabolic effects of adiposity towards disease [23]. For refugees, this process is accelerated as re-settlement often places at risk individuals in environments where calorie-dense, poor quality diets and reduced opportunities for physical exercise are common [24, 25]. Severe psycho-social and lifestyle stressors further increase the risk of chronic disease [26].

The greatest opportunities to reverse these effects occur during infancy and childhood [27] and the effectiveness of interventions during adulthood is less understood. Interventions such as motivational interviewing and community exercise programmes reduce rates of obesity, diabetes mellitus and hypertension [28]. A single, brief counselling session has previously been found to be remarkably effective in improving lipid profiles [29]. It is unknown whether these findings can be translated to refugee populations. Patients are more likely to discuss diet and exercise modification if their physician speaks the same language. Interventions aiming to improve the metabolic health of refugees are more likely to be successful if they are culturally tailored and facilitated [30].

This was a descriptive study with no comparator group. Therefore, an important limitation of these findings is that we do not know if changes are a result of the dietary counselling given or other factors. Whilst some data was obtained on diet and physical activity these were limited and not comprehensive or detailed enough for us to ascertain whether lifestyle changes could have caused the reduction in BMI seen. Future studies would ideally incorporate validated and more accurate measures of diet and physical activity such as nutritionist obtained food and exercise diaries.

Strengths of the study include the high participation rate (230/230) and retention rate (110/120) in patients who were asked to return for follow-up. These may have been due to the high quality of physician–patient relationships. GPs were well known to the participants with most having attended the same practitioners for long periods. Furthermore, GPs were from similar backgrounds and spoke the same language as most of the participants. These factors are likely to have significantly improved the quality and effectiveness of the counselling delivered in this study. Patients are more likely to discuss diet and exercise modification when their physician speaks the same language as them [31] and language concordance between physicians and their patients have been found to result in better clinical outcomes [32]. Similarly, patients are more likely to adopt beneficial health behaviours when they trust their clinician [33]. Future work to measure and assess the impact of both language concordance and patient trust in their healthcare provider would be invaluable particularly in vulnerable groups such as refugees.

## Conclusions

This study is the first to examine weight gain and metabolic health amongst Afghani refugees in Australia over time. In contradiction to many studies showing that weight gain and metabolic disease occurs rapidly in re-settled refugees, our cohort is remarkable for having stable or decreasing weight and triglyceride levels a median of 6 months after the initial visit. We were not able to test the causes underlying these findings, however this study suggests that brief dietary counselling from culturally concordant providers may result in positive BMI changes. Further research is required to test the efficacy of culturally appropriate, clinician-delivered nutrition and lifestyle counselling such as was used in this study in a randomized controlled trial.

## Supplementary Information

Below is the link to the electronic supplementary material.

Supplementary file1 (PDF 531 KB)

Supplementary file2 (PDF 677 KB)
