# Supplementary material for: Hyperlipidaemia and Weight Amongst Afghani Refugees Attending a General Practice Clinic in Regional Australia
Source: J Immigr Minor Health. 2023 Feb 6;25(3):589–95. doi: 10.1007/s10903-022-01446-1 (PMC10212845; doi:10.1007/s10903-022-01446-1)
Supplement: Supplementary file 1 — Supplementary file1 (PDF 531 KB) [file 10903_2022_1446_MOESM1_ESM.pdf]

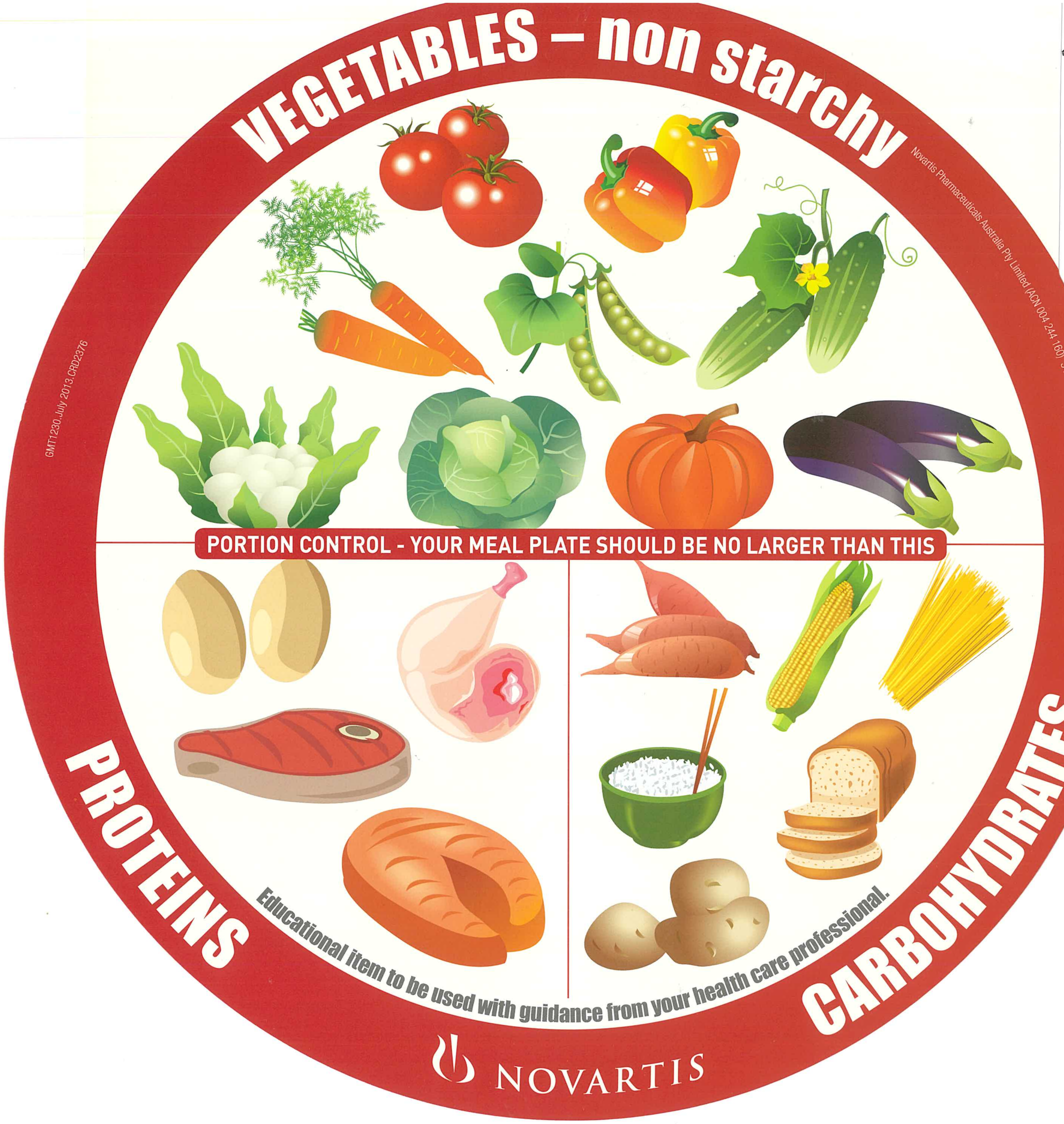

VEGETABLES – non starchy
PORTION CONTROL - YOUR MEAL PLATE SHOULD BE NO LARGER THAN THIS
PROTEINS
CARBOHYDRATES
Educational item to be used with guidance from your health care professional.
NOVARTIS
Novartis Pharmaceuticals Australia Pty Limited (ACN 004 244 160) 54 Waterloo Rd North Ryde NSW 2113 Telephone 02 9805 3555 Fax 02 9805 3751
GMT1230.July 2013.CRD2376
