# Supplementary material for: Hyperlipidaemia and Weight Amongst Afghani Refugees Attending a General Practice Clinic in Regional Australia
Source: J Immigr Minor Health. 2023 Feb 6;25(3):589–95. doi: 10.1007/s10903-022-01446-1 (PMC10212845; doi:10.1007/s10903-022-01446-1)
Supplement: Supplementary file 2 — Supplementary file2 (PDF 677 KB) [file 10903_2022_1446_MOESM2_ESM.pdf]

A GUIDE TO HEALTHY FOOD CHOICES

Healthy eating and regular exercise can help you manage your diabetes as well as other risk factors such as weight, blood pressure, and blood fats. Healthy eating is good for your entire family. It is important to see your GP or dietitian who can provide individual advice about healthy eating for diabetes. Here are some healthy steps you can take that will help in maintaining a healthy lifestyle.

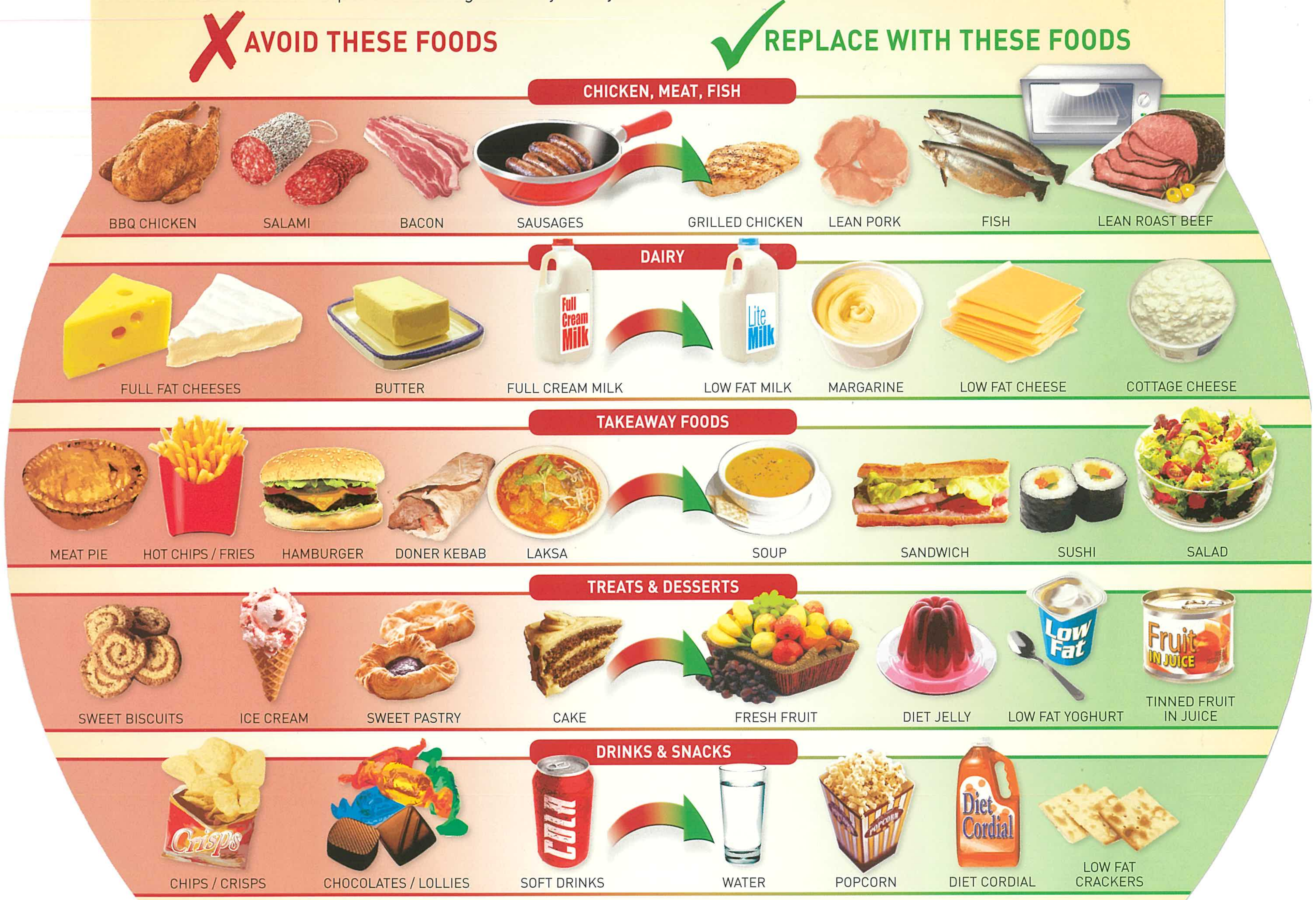

SAMPLE MEAL PLAN

BREAKFAST

Option 1). 2 slices wholegrain bread or toast with ½ cup of beans or spread (e.g. jam, honey or vegemite)

OR

Option 2). Half- one cup of high fibre breakfast cereal with one cup of low fat milk.

LUNCH

Two slices wholemeal bread or one wholemeal bread roll with salad, a small serve of lean meat or skinless chicken or fish and fruit or low fat yoghurt.

SNACKS (if required)

One piece of fruit or 200g tub low fat yoghurt or one slice of wholegrain/fruit bread.

DINNER

A small serve of lean meat, chicken or fish.
One cup cooked low GI rice e.g. basmati or wholemeal pasta or potato or two slices of wholegrain bread.
Lots of salad and/or vegetables.
Fresh fruit or low fat yoghurt.

Adapted from http://swapit.gov.au/ways-to-swap/food-swap-suggester

GMT1230.July 2013.CRD2376.
